# Are the public getting the message about antimicrobial resistance?

**DOI:** 10.1186/s13690-015-0108-6

**Published:** 2015-11-12

**Authors:** Laura J Shallcross, Michelle Berin, Jennifer Roe, Mahdad Noursadeghi

**Affiliations:** UCL Centre for Infectious Disease Informatics, Farr Institute of Health Informatics Research, 222 Euston Road, London, NW1 2DA UK; UCL Infection and Immunity, Cruciform Building, Gower Street, London, WC1E 6BT UK

**Keywords:** Antimicrobial resistance, Patient engagement, Public health, Antimicrobial prescribing

## Abstract

Raising public awareness of the need to use antibiotics appropriately is a major focus of the UK Government’s strategy to tackle antimicrobial resistance. To investigate the public’s views on antibiotic use and resistance we conducted a survey of 120 people as part of patient engagement activities held at University College London Hospital in June 2015.

**Electronic supplementary material:**

The online version of this article (doi:10.1186/s13690-015-0108-6) contains supplementary material, which is available to authorized users.

## Background

Improving public and professional awareness of antimicrobial resistance is a key part of efforts to reduce antimicrobial resistance [1], and is being led by initiatives such as European Antibiotic Awareness Day (EAAD). As part of patient engagement activities at University College London Hospital (UCLH) research open day on 23^rd^ June 2015, we conducted a survey of individuals who were seeking information on research projects related to antibiotic use and antibiotic resistance.

## The study

We developed a one-page survey to assess the public’s attitudes to and knowledge about antibiotic use and resistance, based on a previously validated questionnaire (Additional file 1) [2]. Ethical approval was not sought because the survey formed part of public engagement activities and no personal data was collected. All individuals attending our research stand during the open day were offered the survey. 120 individuals completed the survey, 66 of whom were female. 26 participants were less than 25 years old, 54 were aged 25–44, 25 were aged 45–64 and there were 14 individuals aged over 65 years.

## Findings

Overall 55 % of those surveyed thought the doctor’s decision to prescribe an antibiotic was driven by patient expectation, and this view was most commonly held by individuals aged more than 44 years (74 %). More than two-thirds of those surveyed reported that they trusted the doctor’s decision when he or she chose either to prescribe or to withhold an antibiotic. Most individuals were aware that antibiotics are used to treat bacterial infections (91 %). Of greater concern is the finding that 28 % of individuals thought antibiotics were an effective treatment for viral infections and this view was held by more than one-third of individuals aged less than 25 years. 79 % of individuals completing the questionnaire agreed that resistance to antibiotics is a problem in the UK, but 39 % thought that antibiotic resistance is mainly a problem in other countries. Patients were asked to select the single biggest health problem in the UK from a list comprising: alcohol abuse, cancer, drug abuse, ebola, ‘flu, resistance to antibiotics, smoking and stroke. Cancer (22 %), alcohol abuse (22 %), smoking (18 %) and antibiotic resistance (15 %) were all identified as important health issues but 26 people (22 %) were unable to select a single health problem from the list.

Amongst patients completing our survey there was a high-level of awareness and interest in antimicrobial resistance. This is perhaps unsurprising, given that individuals attending patient engagement events tend to be interested in research and topical health issues. Nonetheless, even within this population, more than one–quarter of individuals thought antibiotics were used to treat viral infections. If we hope to reduce inappropriate antibiotic use, more needs to be done to understand how to communicate messages about appropriate antibiotic use to the public.

## Additional file

Additional file 1:
**Antibiotic questionnaire.**
