# Supplementary material for: Are the public getting the message about antimicrobial resistance?
Source: Arch Public Health. 2015 Nov 12;73:55. doi: 10.1186/s13690-015-0108-6 (PMC4642666; doi:10.1186/s13690-015-0108-6)
Supplement: Additional file 1: — Antibiotic questionnaire. [file 13690_2015_108_MOESM1_ESM.docx]

What is all the fuss about antibiotics- give us your views

Thank you for completing this questionnaire. Please tick the *single* best answer.

| 1. Are you: | | | Male ❑ | | | Female ❑ | | | |
| --- | --- | --- | --- | --- | --- | --- | --- | --- | --- |
| 1. How old are you? | | | 16-24 ❑ | 25-44 ❑ | | | 45-64 ❑ | | Over 65 ❑ |
| 1. Which is the biggest health problem in the UK? | | | Alcohol abuse ❑  Cancer ❑  Drug abuse ❑  Ebola ❑ | | | Flu ❑  Resistance to antibiotics ❑  Smoking ❑  Stroke ❑ | | | |
| 1. Doctors often prescribe antibiotics because the patient expects it | | | | | | | | | |
| Strongly agree | Agree | No opinion | | | Disagree | | | Strongly disagree | |
| 1. I trust the Doctor’s decision when he/she prescribes an antibiotic | | | | | | | | | |
| Strongly agree | Agree | No opinion | | | Disagree | | | Strongly disagree | |
| 1. I trust the Doctor’s opinion when he/she does not prescribe an antibiotic | | | | | | | | | |
| Strongly agree | Agree | No opinion | | | Disagree | | | Strongly disagree | |
| 1. Antibiotics are used to treat infections caused by bacteria | | | | | | | | | |
| Strongly agree | Agree | No opinion | | | Disagree | | | Strongly disagree | |
| 1. Antibiotics are used to treat infections caused by viruses | | | | | | | | | |
| Strongly agree | Agree | No opinion | | | Disagree | | | Strongly disagree | |
| 1. Resistance to antibiotics is a problem in the UK | | | | | | | | | |
| Strongly agree | Agree | No opinion | | | Disagree | | | Strongly disagree | |
| 1. Resistance to antibiotics is mainly a problem in other countries | | | | | | | | | |
| Strongly agree | Agree | No opinion | | | Disagree | | | Strongly disagree | |

Please ask us about what we are doing to prevent threat of antibiotic resistance
